# Effects of Berry Anthocyanins on Cognitive Performance, Vascular Function and Cardiometabolic Risk Markers: A Systematic Review of Randomized Placebo-Controlled Intervention Studies in Humans

**DOI:** 10.3390/ijms22126482

**Published:** 2021-06-17

**Authors:** Sanne Ahles, Peter J. Joris, Jogchum Plat

**Affiliations:** 1 Department of Nutrition and Movement Sciences, School of Nutrition and Translational Research in Metabolism (NUTRIM), Maastricht University, 6200 MD Maastricht, The Netherlands; s.ahles@maastrichtuniversity.nl (S.A.); p.joris@maastrichtuniversity.nl (P.J.J.); 2 BioActor BV, Gaetano Martinolaan 85, 6229 GS Maastricht, The Netherlands

**Keywords:** anthocyanins, cognitive performance, vascular function, cardiometabolic risk markers

## Abstract

Supplementation with anthocyanins, which are a type of flavonoids mainly found in various berries, is hypothesized to be a promising approach to lower the risk of developing cognitive decline. The aim of this systematic review was to provide a comprehensive overview of dietary intervention trials describing effects of berry anthocyanins on cognitive performance in humans, while also addressing potential underlying mechanisms. A total of 1197 articles were identified through a systematic search, and 49 studies reporting effects on cognitive performance (*n* = 18), vascular function (*n* = 22), or cardiometabolic risk markers (*n* = 32) were included. Significant improvements were observed on memory, while some of the studies also reported effects on attention and psychomotor speed or executive function. Vascular function markers such as brachial artery flow-mediated vasodilation were also affected and consistent evidence was provided for the beneficial effects of berry anthocyanins on endothelial function. Finally, studies reported improvements in blood pressure, but effects on metabolic risk markers (e.g. carbohydrate and lipid metabolism) were less consistent. In conclusion, this review provides evidence for the beneficial effects of berry anthocyanins on cognitive performance as memory improved. Whether observed anthocyanin-induced improvements in vascular function and blood pressure underlie beneficial effects on cognitive performance warrants further study.

## 1. Introduction

Cognitive performance encompasses multiple mental abilities that can be categorized into various domains, such as attention and psychomotor speed, memory, and executive function [1]. From childhood, cognitive performance quickly improves until young adulthood, after which it gradually starts declining [2]. Over the next 30 years, the number of people aged 65 years or over is projected to double from 700 million to 1.5 billion worldwide [3]. Therefore, it is becoming increasingly relevant to focus on improving and/or maintaining cognitive performance to delay and prevent cognitive decline, and ultimately the onset of dementia [4]. This could be achieved by targeting potential mechanisms that drive cognitive performance [5,6]. 

An impaired vascular function is a common pathophysiological characteristic of multiple age-related conditions [7,8]. Vascular function can be assessed by determining endothelial function with methods such as brachial artery flow-mediated vasodilation (FMD) or the reactive hyperemia index (RHI) [9]. Furthermore, arterial stiffness can be quantified with measurements such as the augmentation index (AIx) or pulse wave velocity (PWV) [10]. Previous research has already shown that vascular health declines with age leading to an increased risk of cognitive impairment, which may partly be explained by co-existing cardiometabolic risk factors, such as high blood pressure (BP) or a disturbed lipid profile such as altered low-density lipoprotein cholesterol (LDL-C) or high-density lipoprotein cholesterol (HDL-C) concentrations [11]. In fact, the enhancing effect of high BP on cognitive decline is well established [12,13]. Therefore, dietary interventions that target vascular function and/or cardiometabolic risk markers may improve cognitive performance [14,15,16]. 

Anthocyanins are a specific type of flavonoids that are mainly found in various berry fruits (i.e., elderberry, chokeberry, black raspberry, bilberry, blackcurrant, blueberry) [17,18,19], which are known for their health benefits. The main anthocyanin subgroups are cyanidins in elderberry, chokeberry, and black raspberry, delphinidins in bilberry and blackcurrant, and malvidins in blueberry [20]. These anthocyanins have been recognized for protection against cardiovascular and neurodegenerative diseases [21]. Berry fruits, and thereby anthocyanin intake is considered to be low in the Western population [22]. Therefore, increasing dietary intake of anthocyanins through supplementation could be a useful strategy to lower the risk of developing cognitive decline. A recent systematic review by Kent and colleagues [23] reported different intervention studies with beneficial effects of food-derived anthocyanins on cognitive performance. Previous reviews about dietary anthocyanins have mainly focused on the effects of specific fruits, such as blueberries [24,25] and chokeberries [26]. Effects were only evaluated on one specific outcome parameter [27,28], or non-randomized trials were also included in their results [23]. However, a systematic review designed to evaluate the effects of dietary anthocyanin interventions on cognitive performance and underlying mechanisms (i.e., vascular function and cardiometabolic risk markers) in an integrated manner has not been published yet. Therefore, the aim of this systematic literature review was to provide an overview of dietary intervention trials describing effects of berry anthocyanins on cognitive performance, vascular function, and cardiometabolic risk markers in humans. 

## 2. Results

**Figure 1:**
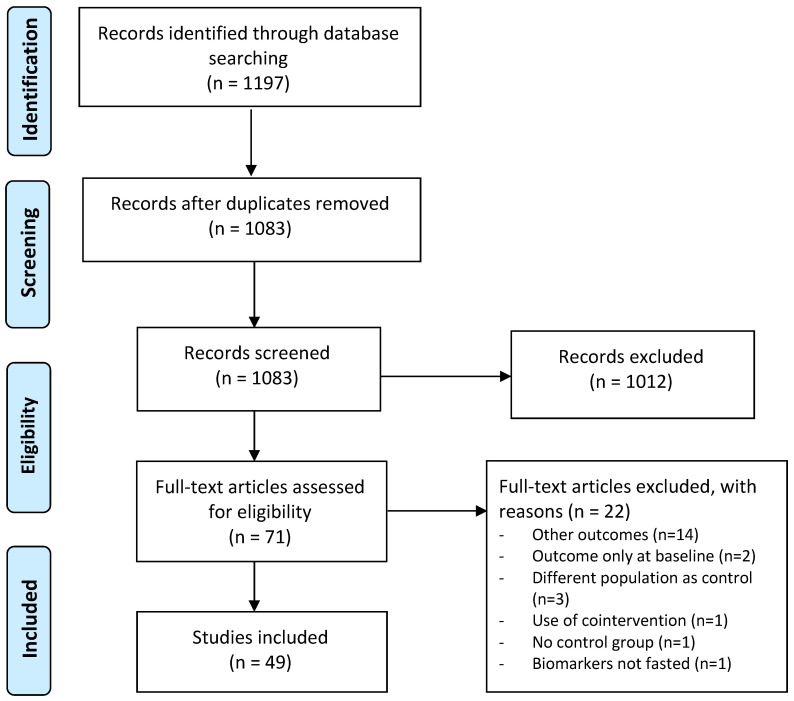
Overview of study selection.

A total of 1197 articles were identified through the systematic search. After removal of duplicates, 1083 articles were screened for inclusion based on title and abstract, which resulted in 71 full-text articles that were considered for inclusion. After detailed reading, 22 articles were excluded for several reasons such as measurement of other outcomes than a priori defined, reporting the correct outcomes but only at baseline, not including a control group, including a different population as a control group, presence of a cointervention, or reporting only unfasted biomarker concentrations. In the end, 49 randomized placebo-controlled intervention studies were included in this systematic review, which enabled us to investigate the effects of berry anthocyanins on cognitive performance, vascular function, and cardiometabolic risk markers. An overview of the study selection is presented in Figure 1.

**Figure 2:**
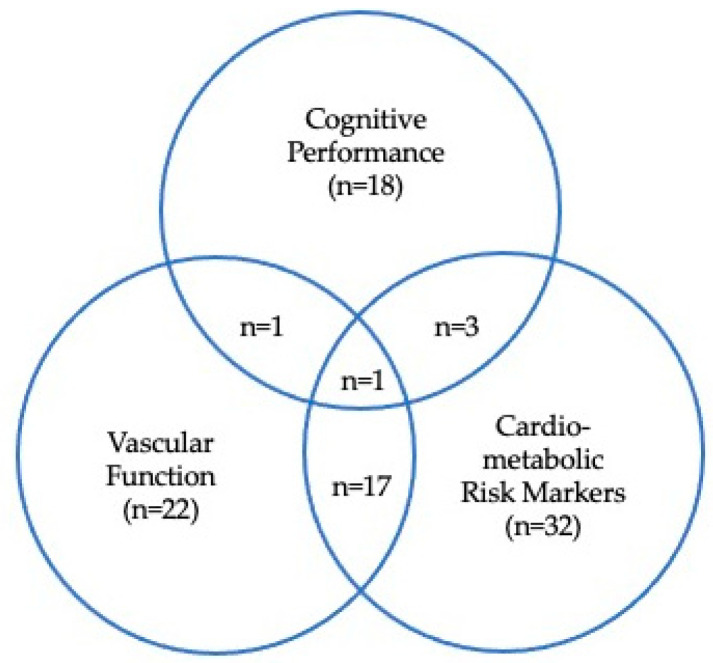
Overview of reported outcomes clustered for cognitive performance, vascular function, and cardiometabolic risk markers in selected articles.

The characteristics of all included studies are shown in Table 1. Two articles [29,30] reported results of two separate studies. In Figure 2, an overview of the reported outcomes is provided. In total, 18 studies reported effects of berry anthocyanins on cognitive performance outcomes. Effects on cardiometabolic risk markers were described in 32 studies, and effects on vascular function were determined in 22 studies. Moreover, three studies reported effects on both cognitive performance and cardiometabolic risk markers, one on cognitive performance and vascular function, and seventeen studies on cardiometabolic risk markers and vascular function. Finally, we performed an intervention trial that addressed, for the first time, anthocyanin-induced effects on cognitive performance and underlying mechanisms (i.e., vascular function and cardiometabolic risk markers) [31]. With the exception of nine articles, the anthocyanin content of the intervention product was provided in all articles and ranged from 1.35–724 mg/day. Study duration varied from 1 h to 24 weeks, and study populations were in general healthy (e.g., children/middle-aged adults). However, a limited number of studies included patient populations suffering from the metabolic syndrome (MetS), myocardial infarction, insulin resistance, or (pre-)hypertension. 

### 2.1. The Effect of Berry Anthocyanins on Cognitive Performance

Of the eighteen studies that determined the effects of berry anthocyanins on cognitive performance outcomes, fifteen used a blueberry intervention, while the other three studies used either a chokeberry extract, a blackcurrant juice, or a blackcurrant extract. Results on cognitive performance were clustered based on the domains evaluated in the studies, i.e., (i) attention and psychomotor speed domain, (ii) executive function domain, (iii) memory domain, or (iv) other tests. Study results are shown in Table 2.

#### 2.1.1. Attention and Psychomotor Speed

Effects of berry anthocyanins on the attention and psychomotor speed domain was evaluated in eight studies. Studies reporting effects of blueberry interventions on the trail-making test part A (TMT-A) are not in accordance. One study in older adults with mild cognitive impairment (MCI) reported a trend for improved performance [54], while two other studies in older adults with subjective MCI [58] and healthy older adults [59] did not show an effect. Whyte et al. [73] evaluated two dosages of a blueberry intervention and reported increased performance on the modified flanker test (MFT) after supplementation with the higher dose (254 mg anthocyanins). In an early study, our group evaluated the effect of two concentrations of a chokeberry extract and reported improved performance on the grooved pegboard test (GPT) after supplementation with the lower concentration (16 mg anthocyanins), but not with the higher concentration (27 mg anthocyanins) [31]. Scores on the five-choice reaction time task (FCRTT) were improved after supplementation with a blackcurrant juice in healthy young adults [71] but not with a blackcurrant extract in healthy older adults [41]. Both studies did not report any changes in cognitive performance on the simple reaction time task (SRTT). Other tests that were carried out in the remaining studies are the attention matrices test (AMT), the number cross-out test (NCT), the rapid visual information processing (RVIP), and the digit vigilance test (DVT). Blueberry juice improved scores on the AMT in middle aged adults [70]. For the other tests, no significant effects were observed [31,41,71].

#### 2.1.2. Executive Function

Effects of berry anthocyanins on the executive function domain were reported in fourteen studies. The most used test was the (modified) attention network task ((M)ANT), which was used in six studies [30,33,59,74,75,76]. Two of these studies reported significantly improved scores after supplementation with blueberries [33,74], and one study supplementing with blueberry powder reported a trend for an improved score [30]. The other studies did not observe an effect of supplementation on the (M)ANT. Four studies reported outcomes of the trail-making test part B (TMT-B), of which one found a significant improvement after post-operative supplementation with blueberry juice [70]. In the other studies, no significant changes for the TMT-B were reported [54,58,59]. Studies that used the Stroop test did not observe any changes [31,36,72,75]. The Go-no-Go test was used in 3 studies by the same research group. In a study in healthy middle aged adults, blueberry supplementation resulted in improved scores [76]. A study in healthy children observed a trend for deteriorated performance in the low dose group compared to placebo [73], while the other study in healthy children reported no change [72]. Other included tests were the controlled oral word associated test (COWAT), spatial working memory test (SWM), task switching test (TST), Stop-Go test, and picture matching task (PMT). For these tests, one study in older adults with MCI found improved scores on COWAT [54], while another in older adults with subjective MCI did not [58], even though the dosages were similar. Furthermore, for the TST, an improvement was reported in heathy older adults [59], but not in healthy children [30]. No changes in the SWM [41] or the Stop-Go test [30] were observed in any of the studies.

#### 2.1.3. Memory

Effects of dietary berry anthocyanins on the memory domain were evaluated in fourteen studies, using a wide variety of tests. Three variations of the verbal learning test were reported. For the Rey auditory verbal learning test (RAVLT), three out of six studies observed improved performance after supplementation with blueberries [33,73,76]. The three other studies reported no effects of blueberry supplementation [30,75], or mixed findings within the test [72]. The California verbal learning test (CVLT) was used in two studies that both reported improved performance after supplementation with blueberry interventions [53,59]. The Hopkins verbal learning test (HVLT) was also used in two blueberry intervention studies, of which one reported an improvement [58], and one did not [54]. Furthermore, two variations on paired associates learning were reported. In two blueberry intervention studies, scores for verbal paired associates learning (VPAL) [53] and spatial paired associates learning (SPAL) improved [54], while SPAL remained unchanged in a study with a blackcurrant extract [41]. One study reported results of the word recognition task (WRT). Here, a dose-dependent effect was observed, with a significant improvement only after supplementation with the highest anthocyanin dose (7 mg) [75]. The n-back test was used in three studies, of which one reported an improvement after blueberry supplementation in healthy older adults [36]. Boespflug et al. [35] observed a trend for improvement after supplementation in older adults with MCI. In a study supplementing healthy children with a blueberry beverage, no effect on the n-back test outcome was found [72]. An improvement on the Prose memory test was reported after supplementation in middle aged adults scheduled for prostatectomy [70]. Furthermore, a trend for improved scores on the Corsi blocks test (CBT) was observed after supplementation with the highest dose of blueberry anthocyanins (7 mg) [75]. Finally, a study by Whyte et al. [30] reported significant improved performance, on the visuo-spatial grid task (VSGT) after supplementation with a blueberry extract, which indicates an improved working memory. No effects on the international shopping list task (ISLT) [36], the digit span task (DST) [59], the object location task (OLT) [72], the serial subtractions task (SST) [75], the Sternberg memory scanning task (SMST) [75], the Brown Peterson task (BPT) [30], or the picture recognition task (PRT) [30] were observed in any of the studies. 

#### 2.1.4. Other Cognitive Tests

Finally, three studies reported outcomes not part of the attention and psychomotor speed, executive function, or memory domains [33,36,59]. No effects of berry anthocyanin supplementation were observed here.

**Table 2:** The effect of berry anthocyanins on cognitive performance outcomes, compared to control.

Author (Year)	Intervention	Anthocyanin Dose	Attention and Psychomotor Speed					Executive Function					Memory					Other
			TMT-A	MFT	GPT	FCRTT	Miscellaneous	TMT-B	Stroop	(M)ANT	Go-No-Go	Miscellaneous	RAVLT - HVLT - CVLT	VPAL and SPAL	WRT	n-back	Miscellaneous	
Ahles (2020) [31]	Chokeberry extract	16 mg			↑		= (NCT)		=									
		27 mg			=		= (NCT)		=									
Barfoot (2019) [33]	Freeze-dried wild blueberry juice	253 mg								↑			↑ (R)					= (TOWRE-2)
Boespflug (2018) [35]	Freeze-dried blueberry powder	269 mg														↑?		
Bowtell (2017) [36]	Blueberry extract	387 mg							=							↑	= (ISLT)	= (Groton Maze)
Cook (2020) [41]	New Zealand blackcurrant extract	210 mg				=	= (RVIP, SRT)					= (SWM)		= (S)				
Krikorian (2010) [53]	Blueberry juice	428-598 mg ^1^											↑ (C)	↑ (V)				
Krikorian (2020) [54]	Freeze-dried blueberry fruit powder	258 mg	↑?					=				↑ (COWAT)	= (H)	↑ (S)				
McNamara (2018) [58]	Freeze-dried blueberry powder	269 mg	=					=				= (COWAT)	↑ (H)					
Miller (2018) [59]	Freeze-dried blueberry powder	230 mg ^#^	=					=		=		↑ (TST)	↑ (C)				= (DST)	= (VMWMT)
Traupe (2018) [70]	Blueberry juice	nr					↑ (AMT)	↑									↑ (Prose Memory)	
Watson (2019) [71]	Blackcurrant juice	115.09 mg				↑	= (DVT, SRTT)											
Whyte (2015) [72]	Blueberry juice	143 mg							=		=		=? (R)			=	= (OLT)	
Whyte (2016) [73]	Freeze-dried wild blueberry powder	127 mg		=							↓?	↑? (PMT)	↑? (R)					
		254 mg		↑							=	↑? (PMT)	↑ (R)					
Whyte (2017) [74]	Wild blueberry powder	253 mg								↑								
Whyte (2018) [75]	Wild blueberry powder and extract	1.35 mg							=	=			= (R)		=		= (CBT, SST, SMST)	
		2.7 mg							=	=			= (R)		=		= (CBT, SST, SMST)	
		7 mg							=	=			= (R)		↑		↑? (CBT); = (SST, SMST)	
Whyte (2020) [76]	Wild blueberry powder	475 mg								=	↑		↑ (R)					
Whyte (2020) [30]	Wild blueberry powder	253 mg											= (R)				↑ (VSGT); = (BPT, PRT)	
	Wild blueberry powder	253 mg								↑?		= Stop-Go, TST)						

↑ or ↓ or = indicates statistically significant improved or deteriorated values or no significant change in the intervention group compared to control. ? indicates a trend. # indicates that the value was calculated; ^1^ indicates that the dosage was dependent on body weight. Abbreviations: AMT: attention matrices test; BPT: brown peterson task; CBT: Corsi blocks test; COWAT: controlled oral word association; CVLT: california verbal learning test; DST: digit span task; DVT: Digit vigilance test; FCRTT: five-choice reaction time task; GPT: grooved pegboard test; HVLT: hopkins verbal learning test; ISLT: international shopping list task; MANT: modified attention network task; MFT: modified flanker test; nr: not reported; OLT: object location task; PMT: picture matching task; NCT: number cross out test; PRT: picture recognition task; RAVLT: rey auditory verbal learning test; RVIP: rapid visual information processing; SMST: sternberg memory scanning task; SPAL: spatial paired associates learning; SRTT: simple reaction time task; SST: serial subtractions task; SWM: spatial working memory task; TMT: trail making test; TOWRE-2: test of word reading efficiency; TST: task switching test; VMWMT: Virtual Morris Water Maze test; VPAL: verbal paired associates learning; VSGT: visuospatial grid task; WRT: word recognition task.

### 2.2. The Effect of Berry Anthocyanins on Vascular Function

Twenty-two studies reported outcomes on vascular function markers. The interventions used in these studies were blueberry (*n* = 11), blackcurrant (*n* = 6), black raspberry (*n* = 3), and chokeberry (*n* = 2). The results were clustered based on the type of measurement i.e., (i) FMD, (ii) RHI, (iii) AIx, (iv) PWV, and (v) other measurements. The results of all studies are displayed in Table 3 and Table S1.

#### 2.2.1. Brachial Artery Flow-Mediated Vasodilation

Effects of berry anthocyanins on FMD was evaluated in eight studies. Black raspberry supplementation in adults with MetS significantly increased FMD [47]. In addition, six studies evaluated the effect of multiple anthocyanin concentrations on FMD. Supplementation with freeze-dried blueberry powder in adults with MetS [43] and blackcurrant juice in healthy adults [52] improved FMD in a dose-dependent manner. In the other studies, all carried out in healthy men, all tested concentrations increased FMD [29,46,66]. However, Tomisawa et al. [69] reported that FMD remained unchanged after a blackcurrant intervention in young male smokers.

#### 2.2.2. Reactive Hyperemia Index

The reactive hyperemia index (RHI) was measured in four studies. In two of those, a significant increase in RHI was observed in healthy men at cardiovascular risk [65] and adults with MetS [68]. In two studies with a healthy young male population, no changes were observed [44,45].

#### 2.2.3. Augmentation Index

Effects of berry anthocyanins on AIx were evaluated in nine studies. Four studies observed a significant decrease compared to placebo after supplementation. These studies were carried out with a blueberry powder in adults with MetS [43] and sedentary individuals [56], a black raspberry extract in adults with metS [49], or a blackcurrant extract in healthy older adults [62]. In the other five studies, no significant change was observed after supplementation with a black raspberry extract in prehypertensive adults [48], blueberry powder in sedentary individuals [57], blueberry powder or juice in healthy men [29,45]. 

#### 2.2.4. Pulse Wave Velocity

Carotid-to-femoral PWV (cfPWV) was determined in five studies. Supplementation with a blueberry powder in postmenopausal women with (pre)hypertension [51] and a blackcurrant extract in healthy older adults [62] significantly increased cfPWV as compared to the control group. However, cfPWV remained unchanged in healthy men that received a blueberry powder for 24 h [29], and in two short-term blueberry intervention studies in sedentary individuals [56,57]. Moreover, no significant effect of anthocyanin supplementation was observed in the three studies that determined brachial-ankle PWV (baPWV) [47,48,51].

#### 2.2.5. Other Vascular Function Markers

Next to the four measurements for vascular function as mentioned above, several other measures were included (Table S1). A study by Cook et al. [40] observed a significant decrease in total peripheral resistance after supplementation with a blackcurrant extract. The other parameters like carotid artery intima-media thickness (cIMT) or ankle brachial index (ABI) did not change as a result of anthocyanin supplementation.

**Table 3:** The effect of berry anthocyanins on vascular function-related outcomes, compared to control.

Author (Year)	Intervention	Anthocyanin Dose	FMD	RHI	AIx	PWV	
						Carotid-to-femoral	Brachial-ankle
Ahles (2020) [31]	Chokeberry extract	16 mg; 27 mg					
Castro-Acosta (2016) [37]	Blackcurrant extract	131 mg; 322 mg; 599 mg					
Cook (2017) [40]	New Zealand blackcurrant extract	105 mg; 210 mg; 315 mg					
Curtis (2019) [43]	Freeze-dried blueberry powder	182 mg	=		=		
		364 mg	↑		↓		
Del Bó (2013) [44]	Blueberry jello	348 mg		=			
Del Bó (2017) [45]	Blueberry juice	309 mg		=	=		
Istas (2019) [46]	Chokeberry extract and whole fruit	3.6 mg	↑				
		30 mg	↑				
Jeong (2014) [47]	Black raspberry extract	nr	↑				=
Jeong (2016) [48]	Black raspberry extract	nr (low dose)			=		=
		nr (high dose)			=		=
Jeong (2016) [49]	Black raspberry extract	nr			↓		
Jin (2011) [50]	Blackcurrant juice	nr					
Johnson (2015) [51]	Freeze-dried blueberry powder	103 mg ^#^				↓	=
Khan (2014) [52]	Blackcurrant juice	10 mg	=				
		37.75 mg	↑				
McAnulty (2014) [56]	Blueberry powder	nr			↓	=	
McAnulty (2019) [57]	Freeze-dried blueberry powder	nr			=	=	
Okamoto (2020) [62]	New Zealand blackcurrant extract	210 mg			↓	↓	
Riso (2013) [65]	Freeze-dried blueberry powder	375 mg		↑	=		
Rodriguez-Mateos (2013) [29]	Freeze-dried blueberry powder	310 mg	↑		=	=	
		517 mg	↑		=	=	
		724 mg	↑		=	=	
	Freeze-dried blueberry powder	129 mg	↑				
		258 mg	↑				
		310 mg	↑				
		517 mg	↑				
		724 mg	↑				
Rodriguez-Mateos (2014) [66]	Freeze-dried blueberry powder	196 mg	↑				
		339 mg	↑				
Stull (2015) [68]	Freeze-dried blueberry powder	290.3 mg		↑			
Tomisawa (2019) [69]	Blackcurrant extract	50 mg	=				

↑ or ↓ or = indicates statistically significant higher or lower values or no significant change in the intervention group compared to control. # indicates that the value was calculated. Abbreviations: ABI: ankle-brachial index; AIx: augmentation index; FMD: brachial artery flow-mediated vasodilation; nr: not reported; PWV: pulse wave velocity; RHI: reactive hyperemia index.

### 2.3. The Effect of Berry Anthocyanins on Cardiometabolic Risk 

Thirty-two studies determined effects of berry anthocyanins on cardiometabolic risk markers. The interventions used were blueberry (*n* = 12), chokeberry (*n* = 7), blackcurrant (*n* = 6), black raspberry (*n* = 4), elderberry (*n* = 2), and bilberry (*n* = 1). The results were clustered into (i) BP measurements, or (ii) metabolic risk markers. The results of all studies are displayed in Table 4 and Table S2. 

#### 2.3.1. Blood Pressure

Twenty-nine studies evaluated the effect of berry anthocyanins on peripheral BP, of which eight reported lower BP values compared to placebo [34,41,51,56,57,61,62,75]. In four of these studies, both systolic and diastolic BP was decreased after supplementation either with a blackcurrant extract [41], blueberry beverage [34], blueberry powder [51], or chokeberry [61]. The other four studies only showed a significant decrease in systolic BP [56,57,62,75]. The remaining twenty-one studies did not observe an effect on peripheral BP. Five studies also evaluated central BP, of which one using a blackcurrant extract observed decreased central diastolic BP [62]. In four studies, mean arterial pressure (MAP) as a marker of BP levels in the microcirculation was determined. In two studies MAP decreased after supplementation with a blackcurrant extract [40,62]. However, two studies with blueberry powder [51] and a blackcurrant extract [39] did not observe changes in MAP. Two studies measured 24-h ambulatory BP in (pre-)hypertensive adults. Both studies reported mean-, daytime-, and nighttime BP, and one study also reported awake- and sleep BP. The study by Jeong et al. [48] observed lower mean and nighttime systolic BP after supplementation with the higher dose of a black raspberry extract. The other study observed lower daytime diastolic BP, a trend for lower mean diastolic BP and awake BP when supplementing with a black chokeberry extract [55]. Heart rate remained unchanged in all seven studies that evaluated effects of dietary anthocyanins [29,32,39,40,42,46,49].

#### 2.3.2. Metabolic Risk Markers

Effects of berry anthocyanins on fasting plasma glucose and/or insulin concentrations were determined in twelve studies. None of the interventions revealed an effect on fasting glucose [34,42,43,46,55,61,62,63,64,65,67,68] or insulin [43,67,68]. Effects on serum lipids (i.e., triacylglycerol (TAG)) concentrations and lipoproteins (i.e., total cholesterol (TC), HDL-C, and LDL-C) were determined in eighteen studies. Lower TC was reported in three studies [38,47,77], and two studies reported decreased LDL-C concentrations after intervention [38,77]. All other studies did not report any changes in TC or LDL-C concentrations [32,34,42,43,46,52,55,60,61,62,63,64,65,67,68]. None of the studies reported changes in TAG or HDL-C concentrations [32,34,38,42,43,46,47,55,60,61,62,63,64,65,67,68,77]. 

Next to the above-mentioned biomarkers, several ratios (ApoB-ApoA1, HDL/LDL, TAG/HDL, TC/HDL) and other markers (HbA1c, HOMA2-IR, insulin sensitivity, ox-LDL) were measured in some of the included studies. Jeong et al. [47] reported a significantly lower TC/HDL ratio after supplementation with a black raspberry extract. Two studies observed lower ox-LDL concentrations after supplementation with a bilberry powder [32] or a chokeberry extract [61]. Finally, insulin sensitivity was improved in a study by Stull et al. [67] after six weeks of blueberry powder supplementation in adults with MetS. No effect of anthocyanin supplementation was found for the other parameters.

**Table 4:** The effect of berry anthocyanins on cardiometabolic risk markers, compared to control.

Author (Year)	Intervention	Anthocyanin Dose	Blood Pressure					Metabolic Risk Markers								
			SBP/DBP	MAP	Central SBP/DBP	24hr ABP SBP/DBP	Heart Rate	Glucose	Insulin	TC	TAG	HDL-C	LDL-C	non-HDL	ApoA1	ApoB
Ahles (2020) [31]	Chokeberry extract	16 mg	=/=		=/=											
		27 mg	=/=		=/=											
Arevström (2019) [32]	Bilberry powder	90 mg ^#^	=/=				=			=	=	=	=			
Basu (2010) [34]	Freeze-dried blueberry juice	742 mg	↓/↓					=		=	=	=	=			
Castro-Acosta (2016) [37]	Blackcurrant extract	131 mg	=/=													
		322 mg	=/=													
		599 mg	=/=													
Cho (2020) [38]	Black raspberry extract	nr	=/=							↓	=	=	↓	↓	=	↓
Cook (2017) [39]	New Zealand blackcurrant extract	210 mg	=/=	=			=									
Cook (2017) [40]	New Zealand blackcurrant extract	105 mg	=/=	=			=									
		210 mg	=/=	↓			=									
		315 mg	=/=	↓?			=									
Cook (2020) [41]	New Zealand blackcurrant extract	210 mg	↓/↓													
Curtis (2009) [42]	Elderberry extract	500 mg	=/=				=	=		=	=	=	=			
Curtis (2019) [43]	Freeze-dried blueberry powder	182 mg	=/=					=	=	=	=	=	=			
Del Bó (2013) [44]	Blueberry jello	348 mg	=/=													
Del Bó (2017) [45]	Blueberry juice	309 mg	=/=				=									
Istas (2019) [46]	Chokeberry extract and whole fruit	3.6 mg	=/=		=/=		=	=		=	=	=	=			
		30 mg	=/=		=/=		=	=		=	=	=	=			
Jeong (2014) [47]	Black raspberry extract	nr								↓	=	=	=		=	=
Jeong (2016) [48]	Black raspberry extract	nr (low dose)	=/=		=/-	=/=										
		nr (high dose)	=/=		=/-	↓/=										
Jeong (2016) [49]	Black raspberry extract	nr	=/=		=/-		=									
Johnson (2015) [51]	Freeze-dried blueberry powder	103 mg ^#^	↓/↓	=			=									
Khan (2014) [52]	Blackcurrant juice	10 mg	=/=							=						
		35.75 mg	=/=							=						
Loo (2016) [55]	Chokeberry juice and powder	1024 mg	=/=			=/↓?		=		=	=	=			=	=
McAnulty (2014) [56]	Blueberry powder	nr	↓/=													
McAnulty (2019) [57]	Freeze-dried blueberry powder	nr	↓/=													
Murkovic (2004) [60]	Elderberry juice	40 mg								=	=	=	=			
Naruszewicz (2007) [61]	Chokeberry extract	64 mg ^#^	↓/↓					=		=	=	=	=			
Okamoto (2020) [62]	New Zealand blackcurrant extract	210 mg	↓/↓?	↓	=/↓			=			=	=	=			
Petrovic (2016) [63]	Chokeberry juice	nr						=		=	=					
Pokimica (2019) [64]	Chokeberry juice	28.3 mg	=/=					=		=	=					
		113.3 mg	=/=					=		=	=		=			
Riso (2013) [65]	Freeze-dried blueberry powder	375 mg	=/=					=		=	=	=	=			
Rodriguez-Mateos (2013) [29]	Freeze-dried blueberry powder	310 mg	=/=				=									
		517 mg	=/=				=									
		724 mg	=/=				=									
Stull (2010) [67]	Freeze-dried blueberry powder	668 mg	=/=					=	=	=	=	=	=			
Stull (2015) [68]	Freeze-dried blueberry powder	290.3 mg	=/=					=	=	=	=	=	=			
Whyte (2018) [75]	Wild blueberry powder and extract	1.35 mg	=/=													
		2.7 mg	=/=													
		7 mg	↓/=													
Xie (2017) [77]	Chokeberry extract	45.1 mg	=/=							↓	=	=	↓			

↑ or ↓ or = indicates statistically significant higher or lower values or no significant change in the intervention group compared to control. ? indicates a trend. # indicates that the value was calculated; ^1^ indicates that the dosage was dependent on body weight. Abbreviations: ABP: ambulatory blood pressure; ApoA1: apolipoprotein A1; ApoB: apolipoprotein B; DBP: diastolic blood pressure; HDL-C: high-density lipoprotein cholesterol; HR; heart rate; LDL-C: low-density lipoprotein cholesterol; MAP: mean arterial pressure; nr: not reported; SBP: systolic blood pressure; TAG: triacylglycerol; TC: total cholesterol.

## 3. Discussion

This systematic review summarized the effects of berry anthocyanins on cognitive performance, vascular function, and cardiometabolic risk markers. Significant improvements were primarily observed on memory, while some of the studies also reported effects on attention and psychomotor speed or executive function. Vascular function markers were also affected, and it can be concluded that berry anthocyanins predominantly improved vascular endothelial function as measured by FMD. Finally, for cardiometabolic risk markers, studies reported significant effects on BP, but effects on metabolic risk markers (e.g., carbohydrate and lipid metabolism) were less consistent.

Most of the included studies evaluating effects on cognitive performance involved either a young healthy population or older adults (with an increased risk of cognitive decline). Studies measuring cognitive performance in children mostly focused on executive function, while studies in older adults primarily focused on memory tests. For studies in young and middle-aged adults, no specific preference for a specific domain was observed. Regarding memory outcomes, limited evidence was available for children and adults. Most evidence comes from studies involving older adults, which reported improved memory scores after supplementation with berry anthocyanins. The effect on memory was most evident among studies that evaluated individuals with (subjective) MCI. This could be attributed to the fact that there is a bigger window for improvement in older adults as compared with healthy younger adults, as the latter are at the peak of their cognitive abilities, while older adults already experience age-related cognitive decline [4]. The main aspect of memory that was affected was verbal memory, measured with three variations on the verbal learning test. In addition, in the paired associates learning tests, measuring new learning, beneficial effects in populations suffering from MCI were observed. This suggests that even though learning capacity is reduced, it is still possible to improve aspects of memory. Contrary to the memory tests, attention and psychomotor speed tests were primarily carried out in young/middle aged adults. Interestingly, all studies involving an adult population observed significant improvements as a result of supplementation, while all four studies using older adults (with cognitive decline) did not. Similarly, an improved executive function was observed in most studies involving children, but was less evident in (older) adults. These results suggest that the attention and psychomotor speed and executive function domains are better targets for improvement in younger populations. Previously, it has been shown that older adults require more time to finish attention tasks, but are able to maintain similar concentration as compared to younger adults [78]. Most of the tests included in this review reported accuracy scores, which might explain why no improvements could be observed for older adults. Alternatively, physical activity has been linked to cognitive functioning [79], which might be different in the study populations that were included in this review. 

Next to the study population, the duration of the intervention and dose of anthocyanins may also play a role. Beneficial effects on cognitive performance were observed both in acute and longer-term studies. In fact, improved cognitive performance was reported for all three domains in both acute and longer-term studies. For attention and executive function tests, results appear to be stronger in case an acute intervention period was used, while memory outcomes were affected more by longer-term studies. This suggests that the ideal study duration is dependent on the selected cognitive domain.

Regarding the dose, interestingly, beneficial effects on cognitive performance parameters were not necessarily observed in those studies that used the highest amounts of anthocyanins. For example, favorable effects on attention and psychomotor speed were observed in healthy middle-aged adults after supplementation with a chokeberry extract containing 16 mg of anthocyanins [31], but not in healthy older adults after 230.4 mg anthocyanin supplementation using a blueberry powder [59]. This suggests that the effect of the intervention does not only depend on the amount of anthocyanin provided, but could for example also be affected by the composition of the intervention product. Within the studies included in this review, powders were used most often, followed by extracts and juices, with blueberries as the main source. All three compositions had the strongest results on the memory domain, with powder interventions significantly improving memory in five out of seven studies. For the executive function domain, powders also seemed to be the most effective, while extracts did not seem to have an effect. For attention and psychomotor speed, no clear patterns could be observed. 

Since beneficial effects of berry anthocyanin supplementation on cognitive performance were observed, the question is how these effects can be explained mechanistically. Potentially, improvements in vascular function and cardiometabolic risk profiles could play a role in these mechanisms. Regarding the vascular measurements, studies on the effects on vascular function markers were primarily performed in adult populations. Effects of berry anthocyanins on endothelial function were measured by FMD, which is the current non-invasive gold standard approach for the assessment of endothelial function [80], in healthy adults and adults at cardiometabolic risk. Except for one study in smokers, all studies reported an improved FMD. In a recent cross-sectional study, a significant association between FMD and MCI was reported in healthy older adults and older adults with MCI [81]. Csipo et al. [82] observed an association in age-related decline in endothelial function and cognitive decline in older adults. Moreover, Naiberg et al. [83] have already reviewed and established a more specific link for both executive function and working memory with FMD. These results indicate that the effects on memory observed in this review, could potentially be the result of an improved vascular function, with endothelial function as measured by FMD as an important factor. In agreement, the RHI, another measure of endothelial function measuring the reperfusion of limbs, was also improved in adults at cardiometabolic risk, but not in healthy subjects. Besides the effects on markers of endothelial function, some of the studies also focused on arterial stiffness. In fact, for AIx, no significant effects were reported in the studies involving a healthy adult population. However, half of the studies performed in an adult population at cardiometabolic risk, and a single study in healthy older adults, observed an improved AIx. Only a limited amount of the included studies performed cfPWV measurements in (older) adults. No effect was observed in the healthy adult population while cfPWV was improved in adults at cardiometabolic risk and healthy older adults. However, it should be considered that the study duration was only 24 h for the healthy adult population, which is too short to induce structural changes in artery walls that are addressed with cfPWV [84]. 

BP was lowered in several studies that included an adult population at cardiometabolic risk (e.g. (pre)hypertension, MetS, obesity). Studies that were carried out with an older adult population (healthy or subjective MCI) all reported beneficial effects on BP. In contrast, studies evaluating BP effects in healthy adults did not observe any changes. This is in line with our earlier findings on cognitive performance, suggesting that dietary anthocyanins have the most pronounced effects in populations with increased cardiometabolic risk, allowing for improvement by the intervention.

Considering the effects of berry anthocyanins on vascular function and cardiometabolic risk profiles as summarized in this review, the effect of intervention composition (i.e., powder, extract, or juice) and study duration is less clear as compared to the observations for the cognitive domain. Six out of fourteen studies using an extract found significant improvements in BP as compared with three out of ten studies using a powder, and one out of six juice intervention studies. Similarly, all four studies using a powder as intervention reported increased FMD, compared to two out of three studies using an extract. This pattern is also similar for the other parameters. All studies observing a beneficial effect on BP had an intervention period of one to twenty-four weeks, while all four acute studies (<24 h) did not report any significant changes, suggesting that a longer intervention is probably needed to induce effects on BP following the intake of anthocyanins. Taken together, these data suggest that the effect of berry anthocyanins on vascular function and cardiometabolic profiles is not only dependent on the population receiving the intervention, but may also be related to other factors, such as the method of administration, and the duration of intervention. Furthermore, physical activity could affect it. 

A limitation of this review study is that the exact composition of the interventions was not always reported. Moreover, the bioactivity of anthocyanins is known to be dependent on their chemical structure [85]. Even though we were able to report the amount of anthocyanin for most of the studies, more specific components such as anthocyanin subgroups (e.g. cyanidins, delphinidins, malvidins) were often not mentioned. Therefore, it was not possible to compare effects of these anthocyanin subgroups. Besides these subgroups, specific biological effects could also be the result of anthocyanin metabolization [86,87]. Furthermore, only a limited amount of studies using an extract provided information on the method of extraction, which could influence the bioactivity of the anthocyanins [88]. Consequently, we recommend future studies to report information on the chemical composition and extraction methods of the study products. 

In conclusion, this systematic review provides evidence for the beneficial effects of berry anthocyanins on cognitive performance as memory was improved. Vascular endothelial function, as measured by FMD and BP were also affected, and these effects may underlie the observed effects on memory. Future studies should focus on exploring a potential causal link between the beneficial effects on cognitive performance and improvement in vascular function and cardiometabolic risk markers.

## 4. Materials and Methods

### 4.1. Search Strategy

This systematic review was carried out following the Preferred Reporting Items for Systematic Review and Meta-Analyses (PRISMA) checklist. The aim was to summarize the effects of dietary anthocyanin intake on cognitive performance, cardiometabolic risk markers, and vascular function. The databases Medline, Embase, and Cochrane library were searched for articles published until October 2020. The search was based on the six most anthocyanin-rich fruits (i.e., elderberry, chokeberry, black raspberry, bilberry, blackcurrant, blueberry) with the following search string: elderberry* or *Sambucus nigra* or chokeberry* or aronia or black raspberry*or *Rubus occidentalis* or *Rubus coreanus miquel* or bilberry* or *Vaccinium myrtillus* or *Vaccinium uliginosum* or *Vaccinium caespitosum* or *Vaccinium deliciosum* or *Vaccinium membranaceum* or *Vaccinium ovalifolium* or blackcurrant or black currant or *Ribes nigrum* or *Cassis* or blueberry* or *Vaccinium angustifolium* or *Vaccinium sect. cyanococcus* or *Vaccinium corymbosum* AND intervention or RCT or trial.

### 4.2. Study Selection

First, duplicates were removed, and articles were screened based on titles and abstracts by two independent researchers (SA and PJJ), using Rayyan QCRI. The inclusion criteria were: (1) randomized placebo-controlled intervention study in humans; (2) full text available in English; (3) at least one cardiometabolic risk marker (BP, carbohydrate or lipid metabolism), vascular function measurement or cognitive performance parameter was reported, and (4) published in a peer-reviewed journal. Conference abstracts and posters were excluded. After this first selection, the selection procedure was repeated using the full text of the remaining articles. In addition, reference lists of included articles were screened for additional relevant papers. 

### 4.3. Data Extraction

Data from the included articles were extracted into a spreadsheet in Excel consisting of study characteristics (first author, publication year, study design, population, study duration, intervention type), baseline characteristics, cognitive performance outcomes, cardiometabolic risk markers, and vascular function outcomes. All outcomes were extracted for each intervention group separately. A pixel ruler was used if the outcome parameters were only reported in graphs. In addition, within-group changes were calculated in those articles not reporting changes, by subtracting group means before intervention from group means after intervention.

Regarding the cognitive performance parameters, there was a large variety in tests utilized. Therefore, we clustered cognitive test outcomes based on the cognitive domains associated with the tests, as reported in the articles. These clusters were attention and psychomotor speed, executive function, memory, and others. Similarly, for cardiometabolic risk markers, a distinction was made between BP measurements and metabolic risk markers. For vascular function measurements, focus was on markers for endothelial function (i.e., FMD and RHI), arterial stiffness (AIx and PWV), and others.

## Figures and Tables

**Table 1:** Study characteristics.

Author (Year)	Country	Study Design	Study Population	Intervention	Anthocyanin Dose/Day	Study Duration	Sample Size *	Age (Years)	Male (%)	Cognitive Domains	Vascular Function	Cardiometabolic Parameters
Ahles (2020) [31]	NL	Parallel	Healthy middle-aged adults	Chokeberry extract	16 mg; 27 mg	24 weeks	34/35/32	53 ^#^	36 ^#^	Attention and Psychomotor Speed, Executive Function	ABI, cIMT, eP	BP
Arevström (2019) [32]	Sweden	Parallel	Myocardial infarction survivors	Bilberry powder	90 mg ^#^	8 weeks	25/25	67 ^#,$^	84 ^#^			BP, Carbohydrate and lipid metabolism
Barfoot (2018) [33]	UK	Parallel	Healthy children	Freeze-dried wild blueberry juice	253 mg	2 h	29/25	8 ^#^	25 ^#^	Executive Function, Memory		
Basu (2010) [34]	USA	Parallel	Obese individuals with MetS	Freeze-dried blueberry juice	742 mg	8 weeks	25/23	50 ^#^	8 ^#^			BP, Carbohydrate and lipid metabolism
Boespflug (2018) [35]	USA	Parallel	Older adults with MCI	Freeze-dried blueberry powder	269 mg	16 weeks	8/8	78 ^#^	44 ^#^	Memory		
Bowtell (2017) [36]	UK	Parallel	Healthy older adults	Blueberry extract	387 mg	12 weeks	12/14	68 ^#^	50 ^#^	Executive Function, Memory, Other		
Castro-Acosta (2016) [37]	UK	Cross-Over	Healthy men and postmenopausal women	Blackcurrant extract	131 mg; 322 mg; 599 mg	2 h	22	45	59		DVP	BP
Cho (2020) [38]	South Korea	Parallel	Individuals with increased cholesterol	Black raspberry extract	nr	12 weeks	39/38	47 ^#^	25 ^#^			BP, Carbohydrate and lipid metabolism
Cook (2017) [39]	UK	Cross-Over	Healthy men	New Zealand blackcurrant extract	210 mg	1 week	13	25	100		Femoral artery diameter	BP
Cook (2017) [40]	UK	Cross-Over	Male athletes	New Zealand blackcurrant extract	105 mg; 210 mg; 315 mg	1 week	15	38	100			BP
Cook (2020) [41]	UK	Cross-Over	Healthy older adults	New Zealand blackcurrant extract	210 mg	1 week	14	60	86 ^#^	Attention and Psychomotor Speed, Executive Function, Memory		BP
Curtis (2009) [42]	UK	Parallel	Healthy postmenopausal women	Elderberry extract	500 mg	12 weeks	26/26	58 ^#^	0			BP, Carbohydrate and lipid metabolism
Curtis (2019) [43]	UK	Parallel	Overweight/obese adults with MetS	Freeze-dried blueberry powder	182 mg; 364 mg	24 weeks	37/39/39	63 ^#^	68 ^#^		AIx, FMD, PWV	BP, Carbohydrate and lipid metabolism
Del Bó (2013) [44]	Italy	Cross-Over	Healthy men	Blueberry jello	348 mg	1 h	10	21	100		RHI	BP
Del Bó (2017) [45]	Italy	Cross-Over	Healthy men	Blueberry juice	309 mg	2 h	12	24	100		AIx, RHI	BP
Istas (2019) [46]	UK	Parallel	Healthy men	Chokeberry extract and whole fruit	3.6 mg; 30 mg	12 weeks	23/23/20	24 ^#^	100		FMD	BP, Carbohydrate and lipid metabolism
Jeong (2014) [47]	South Korea	Parallel	Adults with MetS	Black raspberry extract	nr	12 weeks	39/38	59 ^#^	47 ^#^		ABI, cIMT, PWV, FMD	Carbohydrate and lipid metabolism
Jeong (2016) [48]	South Korea	Parallel	Prehypertensive adults	Black raspberry extract	nr	8 weeks	15/15/15	57 ^#^	53 ^#^		AIx, PWV	BP
Jeong (2016) [49]	South Korea	Parallel	Adults with MetS	Black raspberry extract	nr	12 weeks	26/25	59 ^#^	45 ^#^		AIx	BP
Jin (2011) [50]	UK	Cross-Over	Healthy adults	Blackcurrant juice	nr	2 h	20	45 ^#^	45 ^#^		vascular reactivity	
Johnson (2015) [51]	USA	Parallel	Postmenopausal women with (pre)hypertension	Freeze-dried blueberry powder	103 mg ^#^	8 weeks	20/20	59 ^#^	0		PWV	BP
Khan (2014) [52]	UK	Parallel	Healthy adults with low fruit intake	Blackcurrant juice	10 mg; 35.75 mg	6 weeks	21/22/21	52 ^#^	67 ^#^		FMD	BP, Carbohydrate and lipid metabolism
Krikorian (2010) [53]	USA	Parallel	Older adults with MCI	Blueberry juice	428-598 mg ^1^	12 weeks	9/7	78 ^#^	31 ^#^	Memory		
Krikorian (2020) [54]	USA	Parallel	Older adults with MCI	Freeze-dried blueberry fruit powder	258 mg	16 weeks	16/21	77 ^#^	46 ^#^	Attention and Psychomotor Speed, Executive Function, Memory		
Loo (2016) [55]	Finland	Cross-Over	Adults with mild hypertension	Chokeberry juice and powder	1024 mg	8 weeks	38	56	37 ^#^			BP, Carbohydrate and lipid metabolism
McAnulty (2014) [56]	UK	Parallel	Sedentary individuals	Blueberry powder	nr	6 weeks	13/12	43 ^#^	nr		AIx, PWV	BP
McAnulty (2019) [57]	USA	Parallel	Sedentary individuals	Freeze-dried blueberry powder	nr	3 weeks	10/12	56 ^#^	32 ^#^		AIx, PWV	BP
McNamara (2018) [58]	USA	Parallel	Older adults with subjective MCI	Freeze-dried blueberry powder	269 mg	24 weeks	19/20	68 ^#^	46 ^#^	Attention and Psychomotor Speed, Executive Function, Memory		
Miller (2018) [59]	USA	Parallel	Healthy older adults	Freeze-dried blueberry powder	230 mg ^#^	90 days	18/19	68 ^#^	32 ^#^	Attention and Psychomotor Speed, Executive Function, Memory, Other		
Murkovic (2004) [60]	Austria	Parallel	Healthy adults	Elderberry juice	40 mg	2 weeks	17/17	29 ^#^	59 ^#^			Carbohydrate and lipid metabolism
Naruszewicz (2007) [61]	Poland	Parallel	Myocardial infarction survivors	Chokeberry extract	64 mg ^#^	6 weeks	22/22	66 ^#^	75 ^#^			BP, Carbohydrate and lipid metabolism
Okamoto (2020) [62]	Japan	Cross-Over	Healthy older adults	New Zealand blackcurrant extract	210 mg	1 week	14	73	43		AIx, PWV, PWV	BP, Carbohydrate and lipid metabolism
Petrovic (2016) [63]	Serbia	Parallel	Young athletes	Chokeberry juice	nr	4 weeks	18/14	18 ^#^	47 ^#^			Carbohydrate and lipid metabolism
Pokimica (2019) [64]	Serbia	Parallel	Individuals at cardiovascular risk	Chokeberry juice	28.3 mg; 113.3 mg	4 weeks	27/28/29	41 ^#^	38 ^#^			BP, Carbohydrate and lipid metabolism
Riso (2013) [65]	Italy	Cross-Over	Healthy men at cardiovascular risk	Freeze-dried blueberry powder	375 mg	6 weeks	18	48	100		AIx, RHI	BP, Carbohydrate and lipid metabolism
Rodriguez-Mateos (2013) [29]	UK	Cross-Over	Healthy men	Freeze-dried blueberry powder	310 mg; 517 mg; 724 mg	24 h	10	27	100		AIx, DVP, FMD, PWV	BP
		Cross-Over	Healthy men	Freeze-dried blueberry powder	129 mg; 258 mg; 310 mg; 517 mg; 724 mg	1 h	11	27	100		FMD	
Rodriguez-Mateos (2014) [66]	UK	Cross-Over	Healthy men	Freeze-dried blueberry powder	196 mg; 339 mg	6 h	10	27	100		FMD	
Stull (2010) [67]	USA	Parallel	Obese, insulin resistant adults	Freeze-dried blueberry powder	668 mg	6 weeks	15/17	52 ^#^	16 ^#^			BP, Carbohydrate and lipid metabolism
Stull (2015) [68]	USA	Parallel	Adults with MetS	Freeze-dried blueberry powder	290.3 mg	6 weeks	23/21	57 ^#^	36 ^#^		RHI	BP, Carbohydrate and lipid metabolism
Tomisawa (2019) [69]	Japan	Cross-Over	Healthy male smokers	Blackcurrant extract	50 mg	2 h	13	22	100		FMD	
Traupe (2018) [70]	Italy	Parallel	Middle-aged adults scheduled for prostatectomy	Blueberry juice	nr	appr. 4 weeks	13/13	67 ^#^	nr	Attention and Psychomotor Speed, Executive Function, Memory		
Watson (2019) [71]	UK	Cross-Over	Healthy young adults	Blackcurrant juice	115.09 mg	2 h	9	23	33 ^#^	Attention and Psychomotor Speed		
Whyte (2015) [72]	UK	Cross-Over	Healthy children	Blueberry juice	143 mg	2 h	14	9	71 ^#^	Executive Function, Memory		
Whyte (2016) [73]	UK	Cross-Over	Healthy children	Freeze-dried wild blueberry powder	127 mg; 254 mg	6 h	21	9	43 ^#^	Attention and Psychomotor Speed, Executive Function, Memory		
Whyte (2017) [74]	UK	Cross-Over	Healthy children	Wild blueberry powder	253 mg	3 h	21	8	52 ^#^	Executive Function		
Whyte (2018) [75]	UK	Parallel	Older adults with subjective MCI	Wild blueberry powder and extract	1.35 mg; 2.7 mg; 7 mg	24 weeks	30/30/31/31	71	39 ^#^	Executive Function, Memory		BP
Whyte (2020) [76]	UK	Cross-Over	Healthy middle-aged adults	Wild blueberry powder	475 mg	8 h	35	51	34 ^#^	Executive Function, Memory		
Whyte (2020) [30]	UK	Cross-Over	Healthy children	Wild blueberry powder	253 mg	3 h	18	8	39 ^#^	Executive Function		
	UK	Cross-Over	Healthy children	Wild blueberry powder	253 mg	75 min	17	9	29 ^#^	Memory, Other		
Xie (2017) [77]	USA	Parallel	Former smokers	Chokeberry extract	45.1 mg	12 weeks	25/24	35 ^#^	49 ^#^			BP, Carbohydrate and lipid metabolism

# indicates that the value was calculated; $ indicates that the median was reported; ^1^ indicates that the dosage was dependent on body weight; * for sample size, first the intervention group (if applicable: lowest to highest anthocyanin dose) was reported, followed by control. Abbreviations: ABI: ankle-brachial index; AIx: augmentation index; BP: blood pressure; nr: not reported. cIMT: carotid artery intima-media thickness; DVP: digital volume pulse; eP: Peterson equivalent; FMD: brachial artery flow-mediated vasodilation; MCI: mild cognitive impairment; PWV: pulse wave velocity; RHI: reactive hyperemia index.

## Supplementary Materials

The following are available online at https://www.mdpi.com/article/10.3390/ijms22126482/s1.
