# Supplementary material for: Effects of Berry Anthocyanins on Cognitive Performance, Vascular Function and Cardiometabolic Risk Markers: A Systematic Review of Randomized Placebo-Controlled Intervention Studies in Humans
Source: Int J Mol Sci. 2021 Jun 17;22(12):6482. doi: 10.3390/ijms22126482 (PMC8234025; doi:10.3390/ijms22126482)
Supplement: Supplementary file 1 [file ijms-22-06482-s001.zip › ijms-1220782-supplementary.pdf]

Supplementary information

Table S1. The effect of berry anthocyanins on other vascular function-related outcomes, compared to control

Author (year)	Intervention	Anthocyanin Dose	cIMT	eP	DVP-SI	DVP-RI	GTN-mediated dilation	ABI	LDI-Acetylcholine	LDI-sodium nitroprusside	Total peripheral resistance
Ahles (2020) [31]	Chokeberry extract	16 mg<br>27 mg	=<br>=	=<br>=				=<br>=			
Castro-Acosta (2016) [37]	Blackcurrant extract	131 mg<br>322 mg<br>599 mg			=<br>=<br>=	=<br>=<br>=					
Cook (2017) [40]	New Zealand blackcurrant extract	105 mg<br>210 mg<br>315 mg									=<br>↓<br>↓
Curtis (2019) [43]	Freeze-dried blueberry powder	182 mg; 364 mg									
Del Bó (2013) [44]	Blueberry jello	348 mg									
Del Bó (2017) [45]	Blueberry juice	309 mg									
Istas (2019) [46]	Chokeberry extract and whole fruit	3.6 mg; 30 mg									
Jeong (2014) [47]	Black raspberry extract	nr	=					=			
Jeong (2016) [48]	Black raspberry extract	nr (low dose)<br>nr (high dose)									
Jeong (2016) [49]	Black raspberry extract	nr									
Jin (2011) [50]	Blackcurrant juice	nr							=	=	
Johnson (2015) [51]	Freeze-dried blueberry powder	103 mg #									
Khan (2014) [52]	Blackcurrant juice	10 mg<br>37.75 mg					=<br>=				
McAnulty (2014) [56]	Blueberry powder	nr									
McAnulty (2019) [57]	Freeze-dried blueberry powder	nr									
Okamoto (2020) [62]	New Zealand blackcurrant extract	210 mg									
Riso (2013) [65]	Freeze-dried blueberry powder	375 mg									
Rodriguez-Mateos (2013) [29]	Freeze-dried blueberry powder	310 mg<br>517 mg<br>724 mg			=<br>=<br>=	=<br>=<br>=					

	Freeze-dried blueberry powder	129 mg; 258 mg; 310 mg; 517 mg; 724 mg
Rodriguez-Mateos (2014) [66]	Freeze-dried blueberry powder	196 mg; 339 mg
Stull (2015) [68]	Freeze-dried blueberry powder	290.3 mg
Tomisawa (2019) [69]	Blackcurrant extract	50 mg

↑ or ↓ or = indicates statistically significant higher or lower values or no significant change in the intervention group compared to control. # indicates that the value was calculated. Abbreviations: ABI: ankle-brachial index; cIMT: carotid intima media thickness; DVP-RI: digital volume pulse reflection index; DVP-SI: digital volume pulse stiffness index; eP: Peterson equivalent; FMD: flow mediated dilation; GTN: glycerol trinitrate; LDI: laser doppler imaging; nr: not reported; PWV: pulse wave velocity; RHI: reactive hyperemia index.

Table S2. The effect of berry anthocyanins on other cardiometabolic risk markers, compared to control.

Author (year)	Intervention	Anthocyanin Dose	Blood Pressure (SBP/DBP)				Metabolic markers							
			Daytime ABP	Night-time ABP	Awake ABP	Sleep ABP	Apo B/ A1 ratio	HbA1c	HOMA2-IR	Insulin sensitivity	ox-LDL	LDL/ HDL	TAG/ HDL	TC/HDL
Ahles (2020) [31]	Chokeberry extract	16 mg; 27 mg												
Arevström (2019) [32]	Bilberry powder	90 mg #						=			↓			
Basu (2010) [34]	Freeze-dried blueberry juice	742 mg						=	=					
Castro-Acosta (2016) [37]	Blackcurrant extract	131 mg; 322 mg; 599 mg												
Cho (2020) [38]	Black raspberry extract	nr									=			
Cook (2017) [39]	New Zealand blackcurrant extract	210 mg												
Cook (2017) [40]	New Zealand blackcurrant extract	105 mg; 210 mg; 315 mg												
Cook (2020) [41]	New Zealand blackcurrant extract	210 mg												
Curtis (2009) [42]	Elderberry extract	500 mg												=
Curtis (2019) [43]	Freeze-dried blueberry powder	182 mg											=	
Del Bó (2013) [44]	Blueberry jello	348 mg												
Del Bó (2017) [45]	Blueberry juice	309 mg												
Istas (2019) [46]	Chokeberry extract and whole fruit	3.6 mg; 30 mg												
Jeong (2014) [47]	Black raspberry extract	nr					=					=	=	↓
Jeong (2016) [48]	Black raspberry extract	nr (lower dose)	= / =	= / =										
		nr (higher dose)	= / =	↓ / =										
Jeong (2016) [49]	Black raspberry extract	nr												
Johnson (2015) [51]	Freeze-dried blueberry powder	103 mg #												
Khan (2014) [52]	Blackcurrant juice	10 mg; 35.75 mg												
Loo (2016) [55]	Chokeberry juice and powder	1024 mg	= / ↓	= / =	↓? / ↓?	= / =	=							=
McAnulty (2014) [56]	Blueberry powder	nr												
McAnulty (2019) [57]	Freeze-dried blueberry powder	nr												
Murkovic (2004) [60]	Elderberry juice	40 mg										=		=
Naruszewicz (2007) [61]	Chokeberry extract	64 mg #									↓			

Okamoto (2020) [62]	New Zealand blackcurrant extract	210 mg		
Petrovic (2016) [63]	Chokeberry juice	nr		
Pokimica (2019) [64]	Chokeberry juice	28.3 mg; 113.3 mg		
Riso (2013) [65]	Freeze-dried blueberry powder	375 mg		
Rodriguez-Mateos (2013) [29]	Freez-dried blueberry powder	310 mg; 517 mg; 724 mg		
Stull (2010) [67]	Freeze-dried blueberry powder	668 mg		↑
Stull (2015) [68]	Freeze-dried blueberry powder	290.3 mg		
Whyte (2018) [75]	Wild blueberry powder and extract	1.35 mg; 2.7 mg; 7 mg		
Xie (2017) [77]	Chokeberry extract	45.1 mg		

↑ or ↓ or = indicates statistically significant higher or lower values or no significant change in the intervention group compared to control. ? indicates a trend. # indicates that the value was calculated; [1] indicates that the dosage was dependent on body weight. Abbreviations: ABP: ambulatory blood pressure; ApoA1: apolipoprotein A1; ApoB: apolipoprotein B; HbA1c: hemoglobin A1C; HOMA2-IR: homeostatic model assessment 2 insulin resistance; DBP: diastolic blood pressure; HDL-C: high-density lipoprotein cholesterol; LDL-C: low-density lipoprotein cholesterol; nr: not reported; ox-LDL-C: oxided low-density lipoprotein cholesterol; SBP: systolic blood pressure; TAG: triacylglycerol; TC: total cholesterol.
